# Antioxidant, anti-inflammatory and protective potential of gallic acid against paraquat-induced liver toxicity in male rats

**DOI:** 10.22038/AJP.2021.18581

**Published:** 2021

**Authors:** Ali Nouri, Najmeh Salehi-Vanani, Esfandiar Heidarian

**Affiliations:** 1 *Department of Biochemistry, Faculty of Medicine, Iran University of Medical Sciences, Tehran, Iran*; 2 *Clinical Biochemistry Research Center, Basic Health Sciences Institute, Shahrekord University of Medical Sciences, Shahrekord, Iran*

**Keywords:** Paraquat, Gallic acid, Inflammation, Liver

## Abstract

**Objective:**

As a herbicide, paraquat is a toxic agent that has devastating effects on human health. Gallic acid, on the other hand, is a natural compound that its anti-oxidant values have been reported in previous studies. Given these, this study was designed to evaluate whether gallic acid could reduce the toxic effects of paraquat in the liver of rats.

**Materials and Methods:**

Six groups of rats were considered in this study. Group 1 (control group), group 2 (25 mg/kg of paraquat), group 3 (paraquat-plus-silymarin), and groups 4, 5, and 6 (paraquat together with gallic acid at the doses of 25, 50, and 100 mg/kg, respectively). After treatment, biochemical, oxidative, and histopathological parameters were evaluated in the rats.

**Results:**

We found that as compared to the control group, while paraquat reduced the hepatic levels of anti-oxidative compounds such as vitamin C (p<0.001), superoxide dismutase (SOD) (p<0.001), and catalase (CAT) (p<0.001), the toxic agent increased the serum levels of protein carbonyl (PC) (p<0.001), malondialdehyde (MDA) (p<0.05), and IL-1β (p<0.001). Paraquat also increased (p<0.05) both serum lipid profile and liver-associated markers in the rats. Nevertheless, gallic acid not only enhanced (p<0.05) the activity of vitamin C, SOD, and CAT but also remarkably reduced (p<0.05) the serum lipid profile, as well as the oxidative and inflammatory markers in the paraquat-treated rats. Gallic acid had also ameliorating effects on the damaged morphology of hepatocytes upon paraquat treatment.

**Conclusion:**

The results of this study suggested that gallic acid possesses reinforcing effects on the antioxidant defense system and could be administered to reduce the toxicity of paraquat.

## Introduction

Paraquat is a poisonous chemical compound that is extensively applied as a plant killer mainly for weed and grass control, particularly in developing countries (Wesseling et al., 2001 ▶). In the bloodstream, a concentration of 8.5 μg/ml paraquat induces toxic effects on the organs such as the heart, liver, lungs, and kidneys (Amin et al., 2020 ▶; Amin et al., 2021b ▶; Winek, 1986 ▶). Although according to the compelling body of evidence paraquat is considered to be toxic for the lungs (Amin et al., 2021a ▶), the toxicity of this compound for other organs, especially the kidney, could be also dreadful. Due to the existence of diverse enzymes which are responsible for regulating cell metabolism and detoxification, it seems that the liver is the main organ for maintaining the balance of the oxidant and antioxidant system. However, hepatocytes are sensitive to xenobiotics, as these compounds could induce oxidative damages in these cells (Gawarammana and Buckley, 2011 ▶). Based on these and according to the U.S. Environmental Protection Agency's Integrated Risk Information System (IRIS), there is a consensus that paraquat could exert oncogenic effects and thereby is a carcinogen (Class C). It has been suggested that paraquat could induce its toxic effects by elevating the production of reactive oxygen species (ROS), which in turn, perturb the redox system and induce oxidative damages. Paraquat toxicity is mainly mediated via oxidative stress-induced mechanisms. Several studies showed that natural bioactive components decline the production of ROS (Karimi-Khouzani et al., 2017 ▶; Nouri et al., 2019 ▶). 

To date, a considerable number of studies have indicated that gallic acid, which could naturally be found in foods, cosmetics, and drugs might have antioxidant properties. Gallic acid is the second most common polyphenolic metabolite that could be found in a wide range of plants (Priscilla and Prince, 2009 ▶). Apart from antioxidant activity, this compound might also exert antibacterial, antiviral, antifungal, and more importantly anticancer effects (Omobowale et al., 2018 ▶). Given these, this study was designed to shed light on the antioxidant activity of gallic acid and demonstrate whether this compound could reduce the toxicity of paraquat in the liver of male rats.

## Materials and Methods

**Chemicals**

Paraquat was purchased from Hamoon Bazr Zarin Co. (Tehran, Iran). 2-Thiobarbituric acid (TBA), sodium acetate, and H_2_O_2_ were procured from Merck Co. (Darmstadt, Germany). Gallic acid, silymarin, nitro blue tetrazolium, TPTZ (2,4,6-tripyridyl-s-triazine), and 2,4‐dinitrophenylhydrazine were obtained from Sigma-Aldrich Co. (St. Louis, MO). SYBR Green Real Time-PCR Master Mix was purchased from the Qiagen Co. (Dusseldorf, Germany). IL-1β kit was prepared from BT Laboratory, China. Total bilirubin, alkaline phosphatase (ALP), alanine aminotransferase (ALT), aspartate aminotransferase (AST), triglyceride (TG), total cholesterol (TC), and low-density lipoprotein cholesterol (LDL-C) kits were purchased from Pars Azmun Co. (Tehran, Iran). All other chemicals applied in this research were of analytical grade.

**Animals and study design**

**Figure 1:**
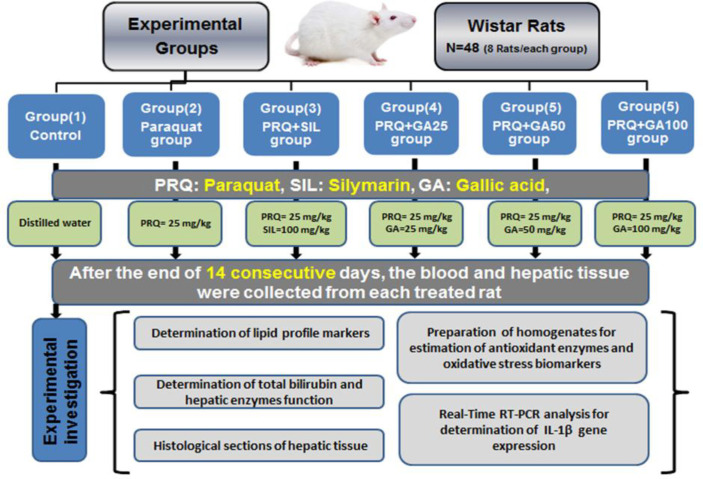
Graphical of study design

To evaluate the efficacy of gallic acid on paraquat-induced toxicity, forty-eight 6–8 week old male Wistar rats with an average weight of 200±20 were obtained from Pasteur Institute (Tehran, Iran). Before initiation of the study, rats could freely have water as well as food and they were kept at 25°C under a 12 hr dark-light cycle. Then, rats were randomly allocated to 6 different groups. Rats in group 1, which was the control group, received 1 ml/kg distilled water by gavage twice daily. Group 2 was orally treated with 25 mg/kg paraquat and an hour later, 1 ml distilled water, which is a solvent of gallic acid (Akinloye et al., 2013 ▶; Sharifi-Rigi et al., 2019 ▶). Group 3 was treated with 25 mg/kg paraquat and 1 ml of silymarin (100 mg/kg po) , as a positive control (Heidarian and Nouri, 2019 ▶). The other three groups were treated with paraquat and an hour later, with gallic acid (25, 50 and 100 mg/kg, po, respectively) (Jadon et al., 2007 ▶; Padma et al., 2011 ▶). After treating all rats with the mentioned agents for 14 consecutive days, the rats which were fasted for 12 hr, ​were sacrificed for collecting the blood sample by cardiac puncture to prepare the serum. Moreover, the liver tissues were also collected from the rats for further histopathological and molecular analyses (Figure 1). Shahrekord University of Medical Sciences Ethics Committee, Shahrekord, Iran has approved this study (Ethic number, IR. SKUMS. REC. 1397. 175). 

**Analyzing the serum biochemical parameters**

The auto-analyzer system (BT3000, Rome, Italy) was applied to evaluate the serum lipid profiles (LDL-C, total cholesterol, VLDL-C, and triglyceride), total bilirubin, ALP, ALT, and AST. Spectrophotometric and Friedewald-equation methods were also used to calculate the value of HDL-C and very-low-density lipoprotein (VLDL), respectively (Friedewald et al., 1972 ▶). 

**Serum antioxidant capacity and liver vitamin C assays**

Ferric reducing/antioxidant power (FRAP) method and 2,4‐dinitrophenylhydrazine reagent were used for assessing serum antioxidant capacity and the activity of vitamin C in the liver of rats, respectively. These assays were well-described in previous studies (Nouri et al., 2019 ▶) (Omaye et al., 1979 ▶). 

**Evaluating the activity of malondialdehyde (MDA) and serum protein carbonyl**

By using the TBA assay, we studied both the serum and the liver levels of MDA in tested rats. The procedure of the assay was according to the study conducted by Heidarian et al. (Heidarian and Soofiniya, 2011 ▶). Moreover, the level of serum protein carbonyl (PC) was evaluated according to Reznick and Packer protocol at 360 nm with 6 M guanidine hydrochloride (Reznick and Packer, 1994 ▶). The results are shown as nmol dinitrophenyl hydrazine (DNPH)/mg protein.

**Assessing the activity of tissue CAT and SOD**

The total protein was extracted from the liver tissue of the rats according to the Bradford assay (Bradford, 1976 ▶). Then, for assessing the liver activity of CAT, we used the protocol described by Heidarian et al. (Heidarian et al., 2014 ▶). Moreover, nitro blue tetrazolium (NBT) was applied to analyze the activity of SOD in the liver of rats. In the presence of SOD, the amount of NBT diminishes and the optical density of the samples could be measured at 560 nm (Flohe, 1984 ▶). Data is shown as U/mg protein. 

**Serum IL-1β analysis**

For studying the serum level of IL-1β, we used the ELISA assay kit according to the manufacturer's instructions. Data is reported as pg/ml. 

**Real-time RT-PCR analysis**

After processing the liver tissues, RNA was extracted using a BIOZOL kit reagent (Bioer, China). The quality and the quantity of the extracted RNA were assessed by Nanodrop ND-1000 instrument (Thermo**, **USA). RNAs whose optical density (OD) 260/280 nm ratio was more than 1.8 were stored for further analysis. The relevant amount of extracted RNAs was then subjected to the reverse transcription reaction to synthesize cDNA using the PrimeScriptTM reagent kit (Takara Bio Inc. Japan). We also designed reverse and forward primers for *IL-1β *and *β-actin, *as an internal control gene, using Oligo 7.0 software. The quality of primers was also confirmed by Blast Nucleotide (NCBI). The sequences of designed primers are shown in Table 1. For studying the alteration in gene expression using the RT-qPCR analysis, the synthesized cDNAs together with primers SYBR^®^ Green PCR Master Mix, and nuclease-free water, were located at a light cycler instrument (Roche). The thermal schedule for the test was as follows: initial activation step of 30 sec at 95°C, and 40 cycles of a denaturation step (15 sec at 95°C), annealing step (20 sec at 60°C), and extension step (25 sec at 72°C). The alteration in gene expression was calculated by using the Livak method by the 2^-ΔΔCt^ formula. 

**Histopathological study**

The liver tissues were fixed in 10% formaldehyde solution and were dissected by a microtome (AMR 400, Amos Scientific, Australia) in 5-μm slices. Each slice was paraffinized and stained with hematoxylin and eosin (H&E) (Carleton et al., 1980 ▶). For histopathological examinations, we used an optical microscope (Nikon Eclipse E400 microscope with digital camera, USA). Then, changes in the tissue morphology were visually assessed within 10 random microscopic fields. The lesions were scored in a blinded manner (Score scale: 0=normal; 1≤25%; 2≤50%; 3≤75%; and 4≤100%).

**Statistical analysis**

The results are representative of three independent tests and are shown as 

mean±SD, except pathological data. For data analysis, SPSS software (Statistical Package for the Social Sciences, version 20.0, SPSS Inc, Chicago, IL) was used. Both one-way analysis of variance (ANOVA) and Tukey's post *hoc* test were used for statistical analysis. A p-value less than 0.05 was considered significant. 

**Table 1:** The list of the primers used in the present study

Gene	Forward primer	Reverse primer
*β-* *actin*	5'-CGCAAATTACCCACTCCCGAC-3'	5'-GTAACCTCCCGTTCAGACCAC-3'
*IL-β*	5'-CAACAAAAATGCCTCGTGCTG-3'	5'-TCGTTGCTTGTCTCTCCTTGTA-3'

## Results

**Effect of gallic acid on serum lipid profile**

Our results showed that the serum levels of TC, TG, LDL-C, and VLDL-C significantly increased (p<0.001), when rats were orally treated for 14 days with paraquat as compared to the control group (Table 2). In the positive control, we found that silymarin could hamper the effects of paraquat on the serum lipid profile. The rats which were treated with paraquat in combination with silymarin had lower serum levels of mentioned parameters as compared to the rats which were only treated with paraquat (Group 2). More interestingly, gallic acid at the concentrations of 25, 50, and 100 mg/kg was dose-dependently successful to prevent the elevating effect of paraquat on the mentioned parameters. As presented in Table 2, the maximum protective effects of gallic acid on paraquat influence on the serum lipid profile was observed at the doses of 50 and 100 mg/kg, which significantly reduced the serum levels of TC, TG, LDL-C, and VLDL as compared to those rats which were only treated with paraquat (p<0.05). It should be noted that the serum levels of VLDL in rats treated with gallic acid at 25 mg/kg remained unchanged.

**Effect of gallic acid on serum ALT, AST, ALP, and total bilirubin**

In agreement with results obtained for lipid profile level, we found that 14 days of paraquat treatment could elevate the levels of ALT, AST, ALP, and total bilirubin in the serums of rats (p<.001)(Table 2). Silymarin was able to reduce the levels of these liver-related parameters in the rats which were treated with paraquat (Table 2). Of note, gallic acid also exerted protective effects against the influence of paraquat on the liver-related biomarkers. As presented in Table 2, rats which were treated with paraquat in combination with 100 mg/kg of gallic acid had lower ALT, AST, ALP, and total bilirubin values than those which were only exposed to paraquat (p<0.001). Gallic acid at the doses of 25 and 50 mg/kg was unable to significantly diminish the serum levels of liver-associated parameters in paraquat-treated rats. 

**Table 2:** Effects of gallic acid on some serum biochemical parameters

PRQ+GA100	PRQ+GA50	PRQ+GA25	PRQ+SIL	PRQ	Control	Parameters
76±11.1^***^	88±7.7^***^	110.2±9.9^***^	76.7±10.7^***^	163.1±15.8^+++^	76.1±4.7	TG (mg/dl)
86.5±8.8^***^	103.9±11.1^***^	121.4±9.3^**^	97.2±6.4^***^	136.6±6.9^+++^	97.9±6.1	Chol (mg/dl)
22±6.1^***^	44.6±13.1^**^	56±5.9	35.9±7.7^***^	61.8±11.9^+++^	32±6.8	LDL-C (mg/dl)
15.2±2.2^***^	17.6±1.5^***^	22±1.9^***^	15.35±2.1^***^	32.6±3.2^+++^	15.2±0.9	VLDL-C (mg/dl)
36.4±2.9^***^	26.6±2.9^***^	24.6±2.9^***^	32.9±4.3^***^	14.7±3.4^+++^	37.6±2.8	HDL-C (mg/dl)
72.9±16.1^***^	98.4±8.7^***^	130.2±11.6	64.4±12.8^***^	135.9±10.7^+++^	63.6±6.2	ALT (U/L)
153.6±12.5^***^	192±13^***^	210.7±19.4^***^	148.5±20.7^***^	290.9±16.5^+++^	159.5±18.7	AST (U/L)
196.7±17.9^***^	250.4±47^***^	298.9±29.3^***^	191.6±21.2^***^	449.7±47.9^+++^	185.9±17.5	ALP (U/L)
1.11±0.26^***^	1.79±0.30	2.20±0.39	0.84±0.11^***^	2.28±0.71^+++^	0.86±0.07	Total bilirubin (mg/dl)

Data are expressed as mean±SD (n=8). Control: control group; PRQ: paraquat-only administered rats (25 mg/kg); PRQ+SIL: rats treated by paraquat (PRQ) plus silymarin (SIL) (100 mg/kg *po*); PRQ+GA25: rats treated by paraquat (PRQ) plus gallic acid (GA) (25 mg/kg *po*); PRQ+GA50: rats treated by paraquat (PRQ) plus gallic acid (GA) (50 mg/kg *po*) and PRQ+GA100: rats treated by paraquat (PRQ) plus gallic acid (GA) (25 mg/kg *po*).^ +^p<0.05 versus control group, ^++^p<0.01 versus control group,^ +++^p<0.001 versus control group, ^*^p<0.05 versus PRQ treated group,^ **^p<0.01 versus PRQ treated group,^ ***^p<0.001 versus PRQ treated group.

**Effect of gallic acid on serum antioxidant capacity and MDA levels**

To evaluate the effects of paraquat on the anti-oxidant system and the antioxidant capacity of the liver, we then assessed the serum levels of antioxidant capacity. As presented in Table 3, our results showed that as compared to the control group, paraquat not only diminished the serum antioxidant capacity (p<0.001) but also noticeably elevated MDA levels in both liver tissue and the serum (p<0.001). Likewise, gallic acid dose-dependently elevated the serum antioxidant capacity in rats that were treated with paraquat (p<0.05). In agreement, this compound also remarkably increased the serum and the tissue levels of MDA in rats that were simultaneously treated with gallic acid and paraquat (p<0.05) (Table 3). 

**Effect of gallic acid on CAT and SOD activities**

To assess the effect of paraquat on the oxidant and antioxidant system of the liver, we evaluated the activity of hepatic CAT and SOD. Our results showed that rats in group 2, those rats which were only treated with paraquat, had lower CAT and SOD activities as compared to the control group (Table 4). Those rats which were treated with paraquat and silymarin, as the positive control, also showed higher CAT and SOD activities as compared to the rats in group 2. Of note, our results showed that gallic acid was able to significantly prevent the effects of paraquat on the anti-oxidant system of the liver. As indicated in Table 4, rats that were treated with paraquat together with gallic acid doses50 and 100 mg/kg had remarkably higher activities of SOD and CAT as compared to the rats which were only treated with paraquat (p<0.05). 

**Table 3:** Effects of gallic acid on FRAP and MDA levels in the experimental groups

PRQ+GA100	PRQ+GA50	PRQ+GA25	PRQ+SIL	PRQ	Control	Parameters
654.1±44.3^***^	562.7±40.4^***^	527±56.1^***^	675.5±92.8^***^	369.3±38.7^+++^	616.1±52.4	Serum FRAP (μM)
9.80±1.56^***^	13.73±1.77^***^	16.74±1.53^*^	9.25±1.14^***^	19.58±1.30^+++^	8.52±0.57	Serum MDA (nmol/L)
1.76±0.53^***^	2.99±0.58^***^	3.55±0.37^**^	1.50±0.28^***^	5.77±0.84^+++^	1.57±0.19	Liver MDA (nmol/mg protein)

Data are expressed as mean±SD (n=8). Control: control group; PRQ: paraquat-only administered rats (25 mg/kg); PRQ+SIL: rats treated by paraquat (PRQ) plus silymarin (SIL) (100 mg/kg *po*); PRQ+GA25: rats treated by paraquat (PRQ) plus gallic acid (GA) (25 mg/kg *po*); PRQ+GA50: rats treated by paraquat (PRQ) plus gallic acid (GA) (50 mg/kg *po*) and PRQ+GA100: rats treated by paraquat (PRQ) plus gallic acid (GA) (25 mg/kg *po*).^ +++^p<0.001 versus control group, ^*^p<0.05 versus PRQ treated group,^ **^p<0.01 versus PRQ treated group, ^***^p<0.001 versus PRQ treated group.

**Table 4:** Effects of gallic acid on CAT (catalase) activity, SOD (superoxide dismutase) activity, vitamin C level and protein carbonyl content

PRQ+GA100	PRQ+GA50	PRQ+GA25	PRQ+SIL	PRQ	Control	Parameters
195.6±15.6^***^	120.5±20.8^***^	73.3±14.1	159.3±30.8^***^	52.1±7.9^+++^	187±15.8	CAT (U/mg protein)
29±1.5^***^	22.2±2.9^***^	16.6±1.9	30.8±2.5^***^	14.5±1.1^+++^	32.4±3	SOD (U/mg protein)
14.2±1.1^***^	12.4±0.5^***^	9.8±0.9	13.9±1.3^***^	8.9±1.1^+++^	14.3±1.1	Vitamin C (mg/g tissue)
4.8±0.9^***^	7.3±1.3^***^	9.8±1.6	4.7±0.9^***^	11.2±1^+++^	4.4±0.56	Protein carbonyl (nmol NADPH/mg protein)

Data are expressed as mean±SD (n=8). Control: control group; PRQ: paraquat-only administered rats (25 mg/kg); PRQ+SIL: rats treated by paraquat (PRQ) plus silymarin (SIL) (100 mg/kg *po*); PRQ+GA25: rats treated by paraquat (PRQ) plus gallic acid (GA) (25 mg/kg *po*); PRQ+GA50: rats treated by paraquat (PRQ) plus gallic acid (GA) (50 mg/kg *po*) and PRQ+GA100: rats treated by paraquat (PRQ) plus gallic acid (GA) (25 mg/kg *po*). ^+++^p<0.001 versus control group, ^*^p<0.05 versus PRQ treated group,^ **^p<0.01 versus PRQ treated group,^ ***^p<0.001 versus PRQ treated group.

**Effects of gallic acid on liver vitamin C and serum PC**

When we evaluated the effects of paraquat on serum PC content, we found that this toxic agent could noticeably increase PC levels in rats (Table 4). However, gallic acid at the doses of 50 and 100 mg/kg or silymarin at the concentration of 100 mg/kg could remarkably counteract the oxidative effects of paraquat. As presented in Table 4 and as compared to the rats in group 2, the level of PC was significantly reduced in the rats which were treated with paraquat in combination with gallic acid/ silymarin (p<0.05). The same results were also obtained when we evaluated the liver vitamin C levels. While paraquat decreased the level of vitamin C in the liver tissue, both silymarin and gallic acid increased the level of this antioxidant enzyme in paraquat-treated rats (p<0.05) (Table 4). 

**Effect of gallic acid on serum level and gene expression of** **IL-1β**

**Figure 2:**
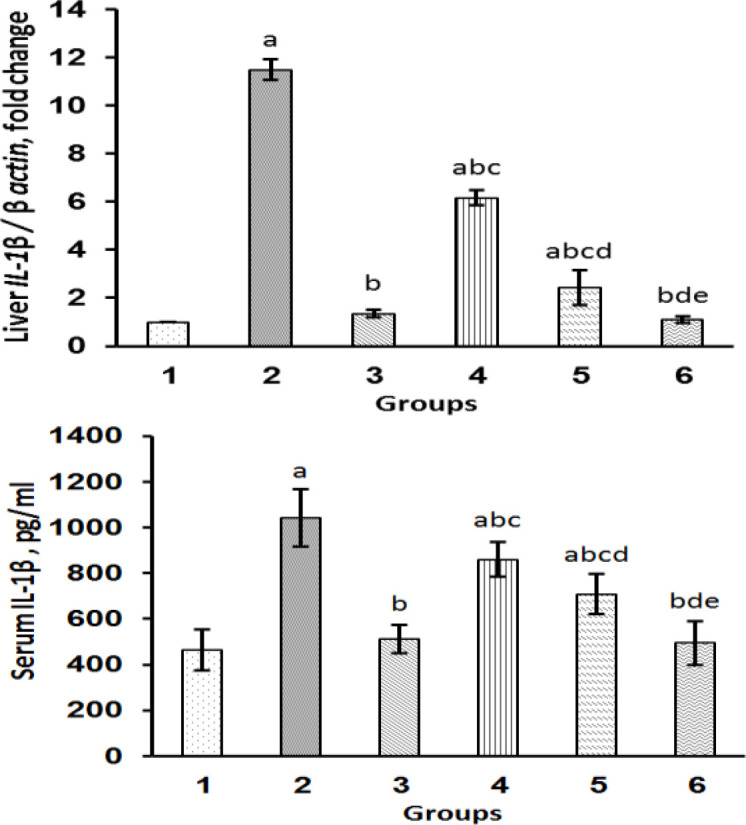
The influence of gallic acid on the serum levels as well as expression of IL-1β. Data are expressed as mean±SD(n=8). Group 1: control group; group 2, paraquat-only administered rats (25 mg/kg); group 3, rats treated by paraquat plus silymarin (100 mg/kg po); groups 4, 5, and 6 were treated by paraquat plus gallic acid (25, 50, and 100 mg/kg po respectively). ^a^p<0.05 versus control group (Group 1).^ b^p<0.05 versus paraquat with its dose (Group 2). ^c^p<0.05 versus group treated with silymarin (Group 3). ^d^p<0.05 versus group treated with gallic acid at dose of 25 mg/kg (Group 4). ^e^p<0.05 versus group treated with gallic acid at dose of 50 (Group 5)

To ascertain whether gallic acid could prevent the toxic effects of paraquat in rats, we also evaluated the effect of both agents on serum and the expression levels of *IL-1β*. Our results showed that paraquat not only elevated the expression of *IL-1β* in the liver tissue of the rats (Group 2) but also significantly elevated the serum levels of this cytokine (Figure 2). On the other hand, the concentrations of 25, 50, and 100 mg/kg of gallic acid and silymarin (100 mg/kg) remarkably reduced the expression of *IL-1β* (Figure 2) as compared to the group 2. Gallic acid at the concentration of 100 mg/kg was more successful in diminishing the expression of *IL-1β* as compared to two other doses. In agreement with the results of the qRT-PCR analysis, both gallic acid and silymarin decreased the serum levels of *IL-1β *as compared to the rats which were only treated with paraquat (Group 2). No significant difference was found in the serum levels of *IL-1β *between the control group and rats which were treated with either 100 mg/kg gallic acid or silymarin.

**Histopathological findings**

**Figure 3:**
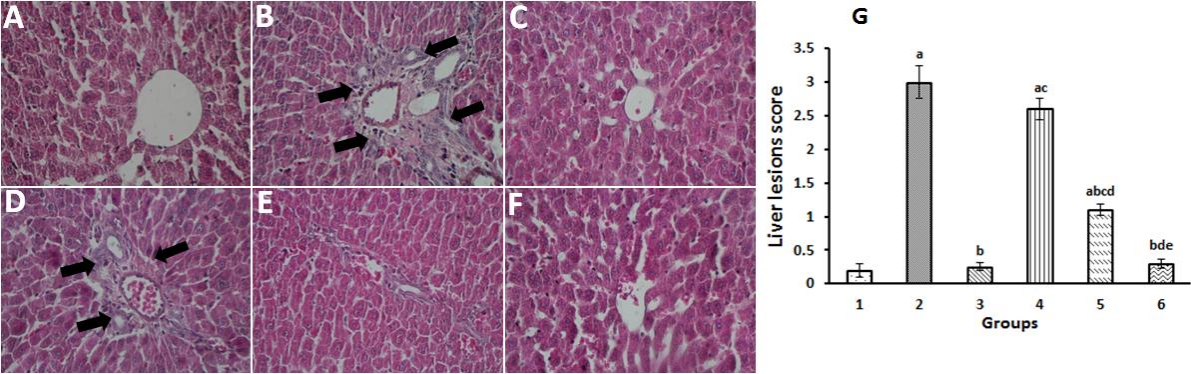
Effects of gallic acid on the liver histology of experimental groups. (A) Control group with normal structure; (B) group 2, paraquat-only administered rats (25 mg/kg); (C) group 3, paraquat-administered rats supplemented with silymarin (100 mg/kg bw); (D) group 4, paraquat-administered rats supplemented with gallic acid (25 mg/kg bw); (E) group 5, paraquat-administered rats supplemented with gallic acid (50 mg/kg bw); (F) group 6, paraquat-administered rats supplemented with gallic acid (100 mg/kg bw). (G) H&E semiquantitative scoring of liver lesions. The black arrows show lymphocyte infiltration

The results of the histopathological analysis are presented in Figure 3. While the normal morphology was observed on the hepatocytes of the control group (Figure 3A) lymphocyte infiltration could be detected in the hepatocytes of the rats which were treated with paraquat (Figure 3B). In contrast to group 2, rats which were treated with paraquat and silymarin had lower inflammatory cell infiltration (Figure 3C). For gallic acid, while this compound at the concentration of 25 mg/kg was not successful to prevent the infiltration of lymphocytes into the hepatocytes (Figure 3D), the concentrations of 50 and 100 mg/kg of the agent noticeably reduced both the percentage of lymphocytes and liver degeneration as compared to the paraquat-treated rats (Figure 3E and 3F). 

## Discussion

The findings of this study demonstrated that oral administration of paraquat induced toxicity in the liver and propagated oxidative stress in male rats. In this study, silymarin was used as a positive control that has revealed ameliorative action against oxidative stress in many human and experimental models in liver injury (Wellington and Jarvis, 2001 ▶). This agent has demonstrated an ameliorative effect against the liver toxicity of paraquat (Ahmad et al., 2013 ▶). The findings of this research display that gallic acid (100 mg/kg), in similarity with silymarin, has a protective impact on toxicity that paraquat could induce in the liver.

In the present study, we found that paraquat could increase the serum levels of 

lipid profile, as revealed by significant elevations in the levels of Chol, TG, LDL-C, and VLDL-C. In accordance with our study, the hyperlipidemic effect of paraquat has been well-studied previously (El-Rahman et al., 2016 ▶; Sharifi-Rigi et al., 2019 ▶) (Table 2). It has been indicated that perhaps the elevating effect of paraquat on TG could be due to either elevation in the serum levels of the inflammatory product or suppression of lipase activity. Moreover, since lipid peroxidation products could interact with LDL receptors, it is reasonable to assume that this mechanism could lead to the elevation of Chol in the rats (Abd El-Rahman et al., 2016 ▶). A considerable number of investigations have demonstrated that natural compounds can diminish the incidence of hyperlipidemia (Karimi-Khouzani et al., 2017 ▶; Sharifi-Rigi et al., 2019 ▶). Interestingly, in the present study, we found that gallic acid might have both antioxidant and antihyperlipidemic properties, as in the presence of this agent, paraquat could not exert its hyperlipidemic effects. Our results showed that gallic acid significantly reduced the serum lipid profile in the paraquat-treated rats.

Another toxic effect that has been observed for paraquat was its devastating effect on the serum level of the liver-associated parameters such as ALT, AST, ALP, and total bilirubin. Previous studies also declared that paraquat could increase the serum levels of indicated markers in both human and animal models (Hafez, 2009 ▶) (Table 2). Of note, we found that both gallic acid and silymarin were successful to diminish the serum levels of ALT, AST, ALP, and total bilirubin in rats that were previously treated with paraquat, suggesting that both agents might have an ameliorating effect on paraquat-induced hepatotoxicity. The study conducted by Karimi-Khouzani et al. also shed light on the protective effects of gallic acid on paraquat-induced toxicity (Karimi-Khouzani et al., 2017 ▶). 

One of the indicators of lipid peroxidation (LPO) is malondialdehyde (MDA). It has been indicated that upon LPO, the serum levels of MDA increased significantly (Gaweł et al., 2004 ▶). Thus far, numerous studies have reported the effect of paraquat on LPO (Sharifi-Rigi et al., 2019 ▶). In accordance, our results also showed that paraquat not only increased the serum levels of MDA in the rats but also reduced FRAP content in the liver tissue of the rats, suggestive of the toxic effects of the agent on the liver. Gallic acid, contrarily, elevated FRAP value and protected the liver of rats from the devastating effects of paraquat, as it also diminished the serum levels of MDA. Although it is early to hazard a conjecture, it could be proposed that the protective effects of gallic acid against paraquat could be due to its ability in scavenging free radicals. 

As the essential enzymes for the antioxidant defense system, both CAT and SOD are necessary to reduce the toxicity of oxygen-free radicals within cells (Wei et al., 2014 ▶). The study conducted by Sharifi-Rigi et al. indicated that paraquat could hamper the activity of a wide range of antioxidant enzymes in the hepatocytes (Sharifi-Rigi et al., 2019 ▶); the inhibitory effect of paraquat on CAT and SOD activity was also noticeable in our study. Interestingly, the anti-oxidant value of gallic acid became more evident when we found that this natural compound could effectively enhance the enzymatic activity of both CAT and SOD in the liver of the rats which were previously treated with paraquat. It has been claimed that gallic acid has extensive effects on the activity of antioxidant enzymes (Karimi-Khouzani et al., 2017 ▶). Given these and based on the suppressive effects of gallic acid on MDA levels, it could be proposed that this natural compound could disturb the oxidative stress and shift the oxidative balance in favor of the anti-oxidant arm. 

There are multiple lines of evidence declaring that excessive production of ROS could lead to protein oxidation, which leads to an increase in the serum levels of protein carbonyl (PC). Based on the role of paraquat in orchestrating oxidative stress, it has been indicated that this agent could also elevate the serum level of PC (Sharifi-Rigi et al., 2019 ▶). The results of our study indicated that gallic acid ceased the oxidative effects of paraquat in the hepatocytes of the rats by diminishing the levels of PC, shedding more light on the free radical scavenging effects of gallic acid. 

When it comes to anti-oxidant as well as scavenging compounds, no name would be sparkled as bright as vitamin C, a non-enzymatic anti-oxidant agent that could hamper lipid as well as protein oxidation (Koekkoek and van Zanten, 2016 ▶). Given the importance of vitamin C in the anti-oxidant defense system, in the present study, it was of particular interest to evaluate the effects of both paraquat and gallic acid on this vitamin. As expected and in total agreement with our previous results, while paraquat significantly reduced the levels of vitamin C in the rats, gallic acid exerted a different effect on this vitamin and increased its level in the liver tissue of the rats. It could be proposed that probably gallic acid counteracts the oxidative effects of paraquat in the liver and improved the morphology of hepatocytes through elevating the levels of vitamin C and thereby not only enhanced the anti-oxidant system but also increased the free radical scavenging properties. 

Apart from the oxidative value of paraquat, it has been suggested that this agent could increase the infiltration of macrophages/monocytes in different tissues and thereby induce tissue damage via producing pro-inflammatory cytokines such as tumor necrosis factor (TNF-α) or IL-1β. IL-1β is also notorious for its devastating influence on the hepatocytes and there is a compelling body of evidence suggesting that this cytokine could induce liver injury (Sultan et al., 2017 ▶). Accordingly, we also found that paraquat not only could elevate the expression of *IL-1β* in hepatocytes but also could increase the serum levels of this cytokine in the rats. On the other hand, gallic acid was successful to reduce both the expression and the serum levels of IL-1β in response to paraquat in the rats, suggesting that gallic acid could reduce the toxicity of paraquat on hepatocytes also through adjusting the inflammatory responses. Upon tissue damage, leukocytes are the first cells that immigrate into the site of injury to propagate inflammation (Ramezannezhad et al., 2019 ▶). Likewise and in agreement with the results of Sharifi-Rigi et al. (Sharifi-Rigi et al., 2019 ▶), the results of our study also revealed the infiltration of lymphocytes to the liver tissue upon paraquat treatment. Interestingly, what elevated the value of gallic acid in the present study was its effects on the inflammatory responses of paraquat, as revealed by the reduction of lymphocyte infiltration in the hepatocytes and the amelioration in the hepatic lesion score. Taken together, the results of the present study introduced gallic acid not only as an antioxidative compound but also as a natural agent that could reduce inflammatory responses within the hepatocytes. 

Herein, we did not assess the impact of paraquat and gallic acid on other oxidative biomarkers and biochemical parameters for example liver immunoexpression of TNF-α, NF-κB, IL 10, caspase‐3, glutathione reductase, glutathione peroxidase, and glutathione‐S‐transferase. Due to the importance of these drivers in the regulation of oxidative stress, we, therefore, propose that their value should be studied in further investigations.

Overall, the data presented in the present study suggested that gallic acid possesses both anti-oxidative as well as anti-inflammatory properties. Given these and based on its safety profile, it could be proposed that this natural compound could be administered to reduce the toxicity of paraquat in the liver cells. 

## References

[B1] Abd El-Rahman N, Kamal El-Dein E, Abd El-Hady A, Soliman SM (2016). Effect of hesperidin on γ-radiation-and/or paraquat herbicide-induced biochemical, hematological and histopathological changes in rats. Pakistan J Zool.

[B2] Ahmad I, Shukla S, Kumar A, Singh BK, Kumar V, Chauhan AK, Singh D, Pandey HP, Singh C (2013). Biochemical and molecular mechanisms of N-acetyl cysteine and silymarin-mediated protection against maneb-and paraquat-induced hepatotoxicity in rats. Chem Biol Interact.

[B3] Akinloye O, Abioye O, Olaojoyetan O, Awosika O, Akinloye D (2013). Dose-dependent effects of paraquat on c-reactive protein, some lipid profile parameters and histology of tissues in male albino rats. Ife J Sci.

[B4] Amin F, Memarzia A, Kazerani HR, Boskabady MH (2020). Carvacrol and Zataria multiflora influenced the PPARγ agonist effects on systemic inflammation and oxidative stress induced by inhaled paraquat in rat. Iran J Basic Med Sci.

[B5] Amin F, Memarzia A, Rad HK, Kazerani HR, Boskabady MH (2021a). Carvacrol and PPARγ agonist, pioglitazone, affects inhaled paraquat-induced lung injury in rats. Sci Rep.

[B6] Amin F, Roohbakhsh A, Memarzia A, Kazerani HR, Boskabady MH (2021b). Immediate and late systemic and lung effects of inhaled paraquat in rats. J Hazard Mater.

[B7] Bradford MM (1976). A rapid and sensitive method for the quantitation of microgram quantities of protein utilizing the principle of protein-dye binding. Anal Biochem.

[B8] Carleton H, Drury R, Wallington E ( 1980). Carleton's histological technique: Oxford University Press.

[B9] El-Rahman A, Kamal El-Dein E, AM AE-H, Soliman SM (2016). Effect of hesperidin on γ-radiation-and/or paraquat herbicide-induced biochemical, hematological and histopathological changes in rats. Pakistan J Zool.

[B10] Flohe L, 1984 Superoxide dismutase assays, Methods in enzymology.

[B11] Friedewald WT, Levy RI, Fredrickson DS (1972). Estimation of the concentration of low-density lipoprotein cholesterol in plasma, without use of the preparative ultracentrifuge. Clin Chem.

[B12] Gawarammana IB, Buckley NA (2011). Medical management of paraquat ingestion. Br J Clin Pharmacol.

[B13] Gaweł S, Wardas M, Niedworok E, Wardas P (2004). Malondialdehyde (MDA) as a lipid peroxidation marker. Wiad Lek.

[B14] Hafez AM (2009). Antigenotoxic activity of melatonin and selenium against genetic damage induced by paraquat. Aust J Basic & Appl Sci.

[B15] Heidarian E, Nouri A (2019). Hepatoprotective effects of silymarin against diclofenac-induced liver toxicity in male rats based on biochemical parameters and histological study. Arch Physiol Biochem.

[B16] Heidarian E, Rafieian-Kopaei M, Khoshdel A, Bakhshesh M (2014). Metabolic effects of berberine on liver phosphatidate phosphohydrolase in rats fed on high lipogenic diet: an additional mechanism for the hypolipidemic effects of berberine. Asian Pac J Trop Biomed.

[B17] Heidarian E, Soofiniya Y (2011). Hypolipidemic and hypoglycemic effects of aerial part of Cynara scolymus in streptozotocin-induced diabetic rats. J Med Plant Res.

[B18] Jadon A, Bhadauria M, Shukla S (2007). Protective effect of Terminalia belerica Roxb and gallic acid against carbon tetrachloride induced damage in albino rats. J Ethnopharm.

[B19] Karimi-Khouzani O, Heidarian E, Amini SA (2017). Anti-inflammatory and ameliorative effects of gallic acid on fluoxetine-induced oxidative stress and liver damage in rats. Pharmacol Rep.

[B20] Koekkoek W, van Zanten AR (2016). Antioxidant vitamins and trace elements in critical illness. Nutr Clin Pract.

[B21] Nouri A, Heidarian E, Amini-Khoei H, Abbaszadeh S, Basati G (2019). Quercetin through mitigation of inflammatory response and oxidative stress exerts protective effects in rat model of diclofenac-induced liver toxicity. J Pharm Pharmacogn Res.

[B22] Omaye ST, Turnbull JD, Sauberlich HE, 1979 Selected methods for the determination of ascorbic acid in animal cells, tissues, and fluids, Methods in enzymology.

[B23] Omobowale TO, Oyagbemi AA, Ajufo UE, Adejumobi OA, Ola-Davies OE, Adedapo AA, Yakubu MA (2018). Ameliorative effect of gallic acid in doxorubicin-induced hepatotoxicity in Wistar rats through antioxidant defense system. J Diet Suppl.

[B24] Padma VV, Sowmya P, Felix TA, Baskaran R, Poornima P (2011). Protective effect of gallic acid against lindane induced toxicity in experimental rats. Food Chem Toxicol.

[B25] Priscilla DH, Prince PSM (2009). Cardioprotective effect of gallic acid on cardiac troponin-T, cardiac marker enzymes, lipid peroxidation products and antioxidants in experimentally induced myocardial infarction in Wistar rats. Chem Biol Interact.

[B26] Ramezannezhad P, Nouri A, Heidarian E (2019). Silymarin mitigates diclofenac-induced liver toxicity through inhibition of inflammation and oxidative stress in male rats. J HerbMed Pharmacol.

[B27] Reznick AZ, Packer L ( 1994). Oxidative damage to proteins: spectrophotometric method for carbonyl assay, Methods in enzymology.

[B28] Sharifi-Rigi A, Heidarian E, Amini SA (2019). Protective and anti-inflammatory effects of hydroalcoholic leaf extract of Origanum vulgare on oxidative stress, TNF-α gene expression and liver histological changes in paraquat-induced hepatotoxicity in rats. Arch Physiol Biochem.

[B29] Sultan M, Ben-Ari Z, Masoud R, Pappo O, Harats D, Kamari Y, Safran M (2017). Interleukin-1α and Interleukin-1β play a central role in the pathogenesis of fulminant hepatic failure in mice. PLoS One.

[B30] Wei T, Tian W, Liu F, Xie G (2014). Protective effects of exogenous β-hydroxybutyrate on paraquat toxicity in rat kidney. Biochem Biophys Res Commun.

[B31] Wellington K, Jarvis B (2001). Silymarin: a review of its clinical properties in the management of hepatic disorders. BioDrugs.

[B32] Wesseling C, De Joode BVW, Ruepert C, León C, Monge P, Hermosillo H, Partanen LJ (2001). Paraquat in developing countries. Int J Occup Environ Health.

[B33] Winek C (1986). Drug and chemical blood level data 1986.

